# Epidemiology and Transmission Dynamics of Viral Encephalitides in West Africa

**DOI:** 10.3390/idr15050050

**Published:** 2023-09-05

**Authors:** Olalekan Chris Akinsulie, Ridwan Olamilekan Adesola, Victor Ayodele Aliyu, Ifeoluwa Peace Oladapo, Abdulafees Hamzat

**Affiliations:** 1 College of Veterinary Medicine, Washington State University, Pullman, WA 99163, USA; 2 Faculty of Veterinary Medicine, University of Ibadan, Ibadan 200005, Nigeria

**Keywords:** viral encephalitides, transmission dynamics, epidemiology, One Health

## Abstract

Encephalitis is an inflammation of the brain, often caused by an autoimmune reaction, or in most cases because of a direct viral, bacterial, or parasitic infection. Viral encephalitides (VE) presents a significant public health concern globally, especially in West Africa. There are more than five hundred known arthropod-borne viruses (arboviruses), with over a hundred of them identified to cause encephalitic diseases in humans and animals, giving rise to a tremendous burden of the diseases and socioeconomic strains in tropical and subtropical regions worldwide. Despite their importance, few effective preventive and control measures in the form of vaccines and therapies are available, and when they are, their use is limited. These limitations are largely hinged on the paucity of information about the molecular epidemiology and transmission patterns of VE in West Africa. Here, we reviewed the transmission dynamics, molecular epidemiology, and the ecological drivers of VE in West Africa. Collectively, timely and accurate interventions are essential for encephalitic viral disease control. Moreover, the integrated health system approach, combining surveillance, vaccination, vector control, and community engagement, could be effective in preventing viral encephalitis globally.

## 1. Introduction

**Figure 1:**
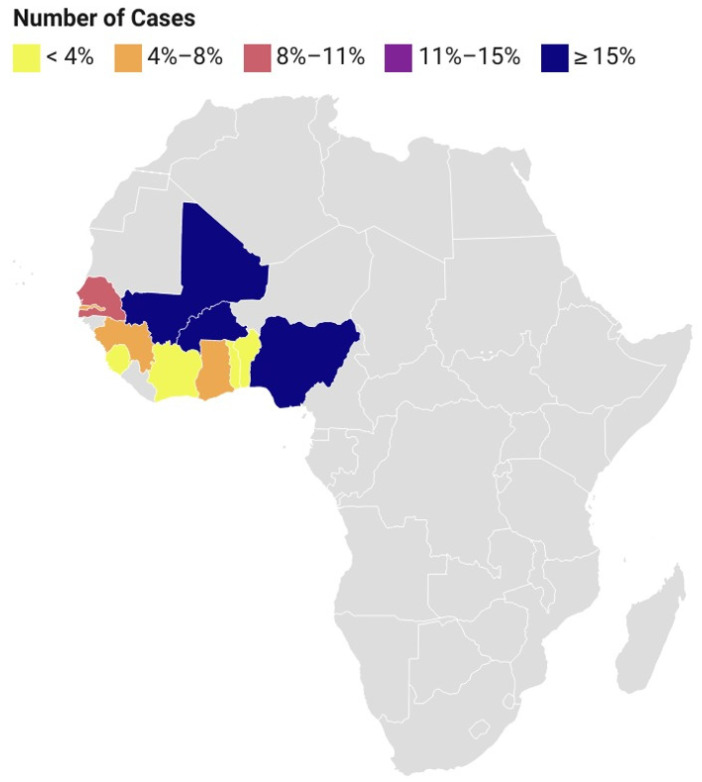
**Map of Africa showing the number of viral encephalitides cases in West African countries (Benin, Burkina Faso, Ghana, Guinea, Ivory Coast, Mali, Nigeria, Senegal, Sierra Leone, The Gambia, and Togo) between 2014–2022.** Six arboviruses causing encephalitis, such as West Nile virus (WNV), Japanese encephalitis virus (JEV), Zika virus (ZIKV), chikungunya virus (CHIKV), yellow fever virus (YFV), and Rift Valley fever virus (RVFV), and twelve non-arboviruses, such as herpes simplex virus types 1 and 2, varicella zoster virus, enteroviruses, La Crosse virus, St. Louis virus, equine viruses, Powassan virus, Epstein–Barr virus, rabies virus, cytomegalovirus, Ebola virus, and Lassa virus, were considered across seventeen West African countries. *Adapted from* [19].

With numerous emerging and re-emerging zoonotic viral infections that cause serious neurological consequences and have recently gained global attention, viral encephalitides (VE) has become a particularly significant public health concern in West Africa [1,2,3]. It is essential to develop a thorough understanding of the molecular inter-relatedness and the transmission dynamics of these infections in order to properly address future danger. There are several crucial elements which potentiate the establishment and transmission cycle of these plethora of pathogens causing VE generally [4]. Firstly, depending on the particular infection, different viruses that cause encephalitis ideally have arthropod or animal reservoirs [1,5,6]. For instance, arthropod-borne viruses (arboviruses), like chikungunya virus (CHIKV), West Nile virus (WNV), Rift Valley fever virus (RVFV), and yellow fever virus (YFV), predominantly exploit infected animals, including birds, livestock, and wild mammals, as the virus’s amplification hosts [7,8]. Furthermore, zoonotic viruses, like Lassa fever virus (LFV) have particular animal reservoirs, including the Mastomys rat, which helps transmit LFV with the accompanying encephalitis [9]. Ticks and mosquitoes are important carriers of arboviruses, like WNV and CHIKV [10,11]. *Aedes mosquitoes*, especially *Aedes aegypti* and *Aedes albopictus*, are well established carriers of these viruses [11]. In most West African countries (Figure 1), especially Nigeria and Ghana, these mosquitoes typically reproduce primarily in urban and peri-urban environments, where people use artificial water storage containers [12]. By feeding on cattle and animals, ticks, especially those from the genus *Hyalomma*, contribute to the spread of encephalitis-causing viruses, like the Crimean–Congo hemorrhagic fever virus (CCHFV) [13]. Secondly, there is a substantial role humans play in the VE transmission cycle. Through the bites of infected ticks or mosquitoes, which act as hosts for the viruses, humans get infected [14]. Working in the veterinary, agricultural, or health sector exposes certain occupational groups to an increased risk of contracting zoonotic viruses, such as LFV, through contact with body fluids [15]. Thirdly, environmental factors are also important in the transmission dynamics of VE. Peak transmission occurs during the rainy season, when mosquito populations are at their highest, and climate and seasonality have a significant impact on tick and mosquito populations [16]. Rapid urbanization and changes in land use encourage mosquito breeding, which amplifies the spread of viruses that cause encephalitis. The spread of *Aedes* mosquitoes in urban settings is further aided by poor waste management procedures [17]. Alongside vector-borne viruses, other encephalitic viruses include enteroviruses and lyssaviruses. Currently, there are no specific treatments or vaccine options for most of these encephalitic viruses; hence, infected humans and animals often require symptomatic treatments. Generally, successful implementation of targeted remedies and control strategies requires an understanding of the intricate drivers of VE transmission. For example, in the case of Ebola virus, a previous study has shown that the effectiveness of an intervention that reduces transmission to susceptible health care workers and increases the sanitary burial of hospital cases is most sensitive and elastic to specific factors, such as the incubation period of the virus, the duration between symptoms, and the onset of mortality [18]. Here, we proposed an effective and unbiased application of the One Health concept to bolster existing preventive and control strategies in West African nations.

## 2. Transmission Dynamics of Major Viral Encephalitides in West Africa

Per the epidemiological triad, three factors are normally required for the transmission, maintenance, and propagation of most infectious diseases [20]: the pathogen (virus), the vector (mosquito or tick, for example), and a suitable virus-replicating host (such as a bird or monkey). All three factors must have adequate and frequent interactions within the environment for the virus to survive and make progress along the next pathway in the cycle; otherwise, local infection and, eventually, the species of the virus will die off. Consequently, robust and integrated environmental and vector management and control strategies are vital for the curb of the viruses causing encephalitis generally [20]. Oftentimes, the virus finds alternate routes for other direct transmissions, such as directly between people via sperm or blood transfusion, as for Zika virus (ZIKV) [21]. However, this is an exception. Sometimes mosquitoes transfer the virus to a dead-end host, as seen in WNV infections, where humans, who are incidentally infected, play no vital role in the virus’s normal cycle and maintenance but have huge consequences since they cause sickness or death in people [22]. Infected humans do not develop a high enough level of viremia to re-infect other mosquitos, which would allow the environmental spread of the virus and thus increase dissemination opportunity and survival possibilities, which does not lead to further spread [21]. Viral encephalitis can either be caused by arboviruses or non-arboviruses [2,18]. For arboviruses, there are several players involved in their life cycle, and a robust knowledge of these is vital for the development of efficient prevention and control strategies [3]. The maintenance and spread of various viral encephalitic pathogens depend on specific reservoirs in West Africa, as aforementioned [6]. Yellow fever virus is a major viral disease in West Africa [23]. Non-human primates, notably monkeys and apes prevalent in forested settings, have been implicated as the main source of YFV [23]. This virus is acquired by mosquitoes, specifically *Ae. aegypti*, when they feed on infected primates, and it is then transmitted to humans through blood feeding [23]. For ZIKV, there is still much to learn about the viral reservoir in West Africa, even though some evidence points to non-human primates, notably monkeys, as the potential hosts [24]. In addition, as ZIKV can remain in bodily fluids, like semen, and be sexually transmitted, infected people can serve as reservoirs for this virus [25]. Another virus-causing encephalitic disease of concern in West Africa is dengue fever, caused by dengue viruses (DENV). Humans, particularly those who have symptomatic or asymptomatic infections, make up the majority of the viral reservoir for DENV [26]. This virus is spread to more people via mosquitoes, continuing the cycle of transmission. Furthermore, another viral infection that causes encephalitis in West Africa is CHIKV. Although non-human primates and other vertebrates may possibly help maintain CHIKV in some ecological contexts, humans are the virus’ principal reservoir [27]. The presence of CHIKV-infected people in the population allows the virus to survive and spread. *Ae. aegypti* and *Ae. albopictus* mosquitoes, in particular, pick up the virus when they feed on infected people and pass it on to other people when they feed on their blood [27]. There are also other arboviruses in the region that have been largely under-investigated, which have the potential of causing an outbreak in either human or animal populations, like Usutu virus [28].

Previous authors have reported seropositivity to henipavirus in *Eidolon helvum* fruit bats in Ghana [29,30], which was the first evidence of henipavirus infection in Africa, even though *Pteropus* spp. fruit bats are not naturally found in mainland Africa. The identity, phylogeny, and characteristics of the infecting virus (or viruses), which elicited the antibody response to henipavirus, requires further study. Nipah virus, which is transmitted by the same fruit bat, also has the potential of becoming a major concern in this region. There have been evidences of both henipavirus and Nipah virus in pigs and horses in other West African countries, like Nigeria [31]. Furthermore, in West Africa, some non-arboviruses cause encephalitis with serious outcomes; for instance, enteroviruses, such as coxsackieviruses and enterovirus 71. Enteroviruses are largely spread by humans, and humans make up most of the reservoir for these viruses [26]. Person-to-person transmission is a factor in their persistence and spread, especially in crowded settings and among young people [32]. The transmission cycle is further aided by environmental contamination brought on by the virus being shed in feces [19]. Also, Ebola viral hemorrhagic disease (EVD), which was first reported in Guinea, Liberia, and Sierra Leone about a decade ago, has since become a major highlight in regard to major encephalitic disease cases in West Africa [2,33]. Ebola virus was introduced into the human population via contact with the blood, organs, or other bodily fluids of an infected animal. For example, the first human EVD case in the West Africa outbreak was presumably infected via exposure to bats. Aside from bats, EVD has also been reported in individuals exposed to infected wild animals, like chimpanzees, antelopes, and gorillas, in places like Gabon, Cote d’Ivoire, and the Republic of the Congo [34]. Also, rabies caused by rabies virus, a lyssavirus, is a neglected, though preventable, zoonotic encephalitic disease that predominantly affects the most vulnerable populations living in the remote rural areas of resource-limited countries, like the West African countries [35]. There are multiple possible transmission routes for rabies, either via bites or scratches from bats, or through percutaneous bites from infected mammals shedding the virus in their saliva, especially dogs [36]. Currently, most countries in Africa are considered endemic for dog-mediated rabies, with an estimated 20,000 or more deaths annually [37].

## 3. Molecular Epidemiology of Major Viral Encephalitides in West Africa

**Figure 2:**
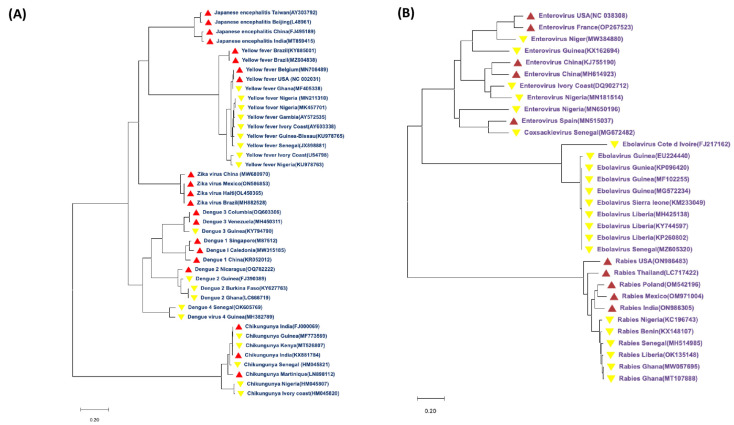
**Phylogenetic trees showing the relatedness of arthropod-borne (A) and non-arthropod-borne (B) viral encephalitides in West Africa and other countries.** Whole genome sequences of the viruses were downloaded from the GenBank across West Africa (yellow triangle) and other countries (red triangle). Countries with partial sequences were not considered, hence excluding several countries from the tree. FASTA files of all the sequences were downloaded and aligned using BioEdit 7.2 software. The phylogenetic tree was constructed using MEGA 11 software. The evolutionary history was inferred using the maximum likelihood method and Tamura–Nei model [40]. For Figure 2A, the tree with the highest log likelihood (−151,004.45) is shown. Initial tree(s) for the heuristic search were obtained automatically by applying the neighbor-join [41] and BioNJ algorithms to a matrix of pairwise distances estimated using the Tamura–Nei model, and then selecting the topology with superior log likelihood value. The tree is drawn to scale, with branch lengths measured in the number of substitutions per site. This analysis involved 41 nucleotide sequences. There was a total of 12,453 positions in the final dataset. Data garnered from the study published by the authors of [38]. For Figure 2B, the tree with the highest log likelihood (−165,598.54) is shown. This analysis involved 32 nucleotide sequences. There was a total of 19,286 positions in the final dataset. Evolutionary analyses were conducted in MEGA11 [42].

A phylogenetic analysis of the sequences of isolated arthropod-borne and non-arthropod-borne viruses that cause encephalitis from various countries in West Africa (Figure 2) shows the genetic evolution and inter-relatedness of these viruses, regardless of their route of transmission [2,38]. Six arthropod-borne viruses causing encephalitis, such as West Nile virus (WNV), Japanese encephalitis virus (JEV), ZIKV, CHIKV, YFV, and RVFV, were considered across seventeen West African countries. Also, 12 non-arthropod-borne viruses, such as herpes simplex virus types 1 and 2, varicella zoster virus, enteroviruses, La Crosse virus, St. Louis virus, eastern and western equine viruses, Powassan virus, Epstein–Barr virus, rabies virus, cytomegalovirus, Ebola virus, and LFV, were considered [38]. Other countries outside West Africa (Taiwan, Beijing, China, Brazil, Belgium, USA, Mexico, Haiti, Columbia, Venezuela, Singapore, Caledonia, Nicaragua, India, and Martinique) were further considered in order to ascertain the evolutionary clustering of these viruses across the West African countries.

The phylogenetic analysis showed a distinct clustering of isolates from the West African countries with other countries, showing evidence that most of these virus-causing encephalitis are endemic and ravaging the African continent. Based on the limited molecular-based network and research in Africa, only a few of these viruses’ molecular signatures are available on GenBank, as evident by the few numbers of countries which have documented the whole genome of the isolated viruses. Only 24 arboviral sequences and 25 non-arthropod-borne viral sequences were available across West Africa based on the findings from this study. There is a need for a robust molecular surveillance network across Africa. Importantly, the level of clustering of these sequences highlights the need for an integrated approach in preventing viral encephalitis across this region [39].

Based on the Tamura 3-parameter model, the evolutionary history of each virus was determined using the maximum likelihood method (MCL) technique [40]. The corresponding taxa’s number of clustered trees are displayed next to the branches. The process of creating the trees involved applying the neighbor-joining [41] and BioNJ algorithms to pairwise distance matrices calculated using the MCL technique [42], and then choosing the topology with the highest log likelihood value. About 1000 repeats of bootstrap values were used to evaluate the phylogenetic groupings’ robustness [42]. With branch lengths calculated as the number of substitutions per site, trees were scaled in their representation.

## 4. Ecological Factors Potentiating the Spread of Viral Encephalitides in West Africa

Environmental and human factors seem to have a role in the rising prevalence of vector-borne illnesses. Changes in the climate, urbanization (particularly in altered urban environments), human actions, large-scale gathering events, human and animal movement, expansion of air travel, and large-scale farming practices have all been implicated in the swiftly expanding world epidemic of vector-borne illnesses [43]. Finally, growing human population density, widespread land use change, and the emergence of human commensal vectors may exert selection pressure on viruses to develop and take advantage of novel habitats [43].

Several of these ecological factors have been briefly overviewed in the following sub-sections.

### 4.1. Climate: Climatic Factors and Climate Change

Changes in the climate, such as increases in temperature and rainfall, can create more favorable conditions for the survival, reproduction, and spread of arthropods, which increase their population and the risk of disease transmission. For example, evidence from the literature suggests that in regions where temperatures are regularly between 25 °C and 29 °C, essentially a greater part of West Africa, a warming climate will become less suitable for malaria, which is endemic in those areas, and more suitable for DENV, CHIKV, YFV, and other arboviruses [44]. Climate change, according to the 2014 Climate Change Report, can promote the propagation of vector-borne infections by modifying the biology of the vectors, their level of abundance and distribution over space, including their territorial expansion into new areas, and changes in the infectious agents’ extrinsic incubation period. Environmental modifications aimed to offset the consequences of climate change, such as flood protection and more urban green space, might raise the risk of illnesses spread by vectors [45].

Ambient temperature has been known for some time to influence viral replication rates and transmission of WNV, influencing the extrinsic incubation period, the seasonal characteristics of the vector mosquito populations, and the regional variability of human cases [46,47,48]. Increased ambient temperatures boost vector population rise [49], shorten the time between blood feeds, and hasten viral development [46,48]. Several experimental discoveries have demonstrated a link between temperature changes and the survival and competency of the WNV mosquito vectors. Although the quantity of precipitation (rain) might alter the disease prevalence patterns, the response may vary across vast geographic regions due to changes in the mosquito vector’s ecology [50]. Above-average rainfall may enhance mosquito numbers as well as the risk for epidemics of diseases [51]. For instance, the frequency of WNV in the tropics should be highest during the rainy season, when mosquitoes are most prevalent [52], but published data on the epidemiology of WNV in the tropical regions are scarce. While precipitation promotes mosquito population emergence in temperate regions [53], increasing rainfall in a humid environment may have minimal effect if undeveloped mosquito habitats are already numerous. Nevertheless, mosquitoes in dry regions, such as the climate of the Mediterranean type, may benefit from more rain, as it increases the number of undeveloped habitats, particularly when heavy rainfall during the spring promotes stagnant reservoirs at the start of the hot season [49]. The ecologies of the numerous possible mosquito vectors of RVFV contribute to the link between these severe rains and epidemic incidents [54]. Over four years, researchers discovered that above-average rainfall for 85–152 days preceded RVF epidemics in seven out of nine locations in South Africa [55]. Human outbreaks caused by CHIKV occur at unpredictable intervals, ranging from three to twenty years, and frequently coincide with extremely wet times. Outbreaks in rural areas are typically on a limited scale and are reliant on sylvatic mosquito numbers, which rise during periods of intense precipitation [56]. In contrast, certain epidemics in coastal East Africa in 2004 were linked to drought and poor socioeconomic development. In these situations, it was thought that the infrequent replenishment of water reserves and mosquito hatching in storage containers near humans potentiate CHIKV transmission [56].

### 4.2. Human Population Growth and Global Travel

The substantial expansion of the human population in West Africa has resulted in greater urbanization and expanding agriculture, potentially creating a new medium for the transmission of disease. For instance, the pattern, trend, and characteristics of urbanization in Nigeria is somewhat interesting. Several cities and towns are growing with a remarkable pace in urbanization at an extraordinary high rate of about 5–10% annually, giving a rapid expansion of Nigerian cities’ area to more than 10-fold their previous point of growth [57]. Particularly in urban settings, the more populous and the closer together they are distributed, the more hosts and easier accessibility available for blood-feeding mosquitoes. Higher population numbers also indicate more garbage, which produces habitats for receptacle breeders, like *Aedes*, or dirty pools and streams for *Culex*, typically in the poorer and less hygienic metropolitan regions where family finances do not afford effective personal mosquito protection. Rapid and huge population expansion is being accompanied by rising urbanization and global migration [58]. Most sub-Saharan African populations are expected to expand twice the current size [59], resulting in greater human mobility into ancient, wooded regions to obtain access to more land and, hence, exposure to the many arboviruses that exist in wild cycles. It is obvious that increased population, greater urbanization, and increased worldwide travel will create a huge opportunity for the transmission and expansion of mosquitoes, viruses, and related arboviral illnesses. Furthermore, the travel and relocation of humans and animals have accelerated the spread of arthropod-borne viruses to new territories. The transnational and intercontinental movement of infectious vector mosquitoes or their eggs are one of the most significant mechanisms for fast geographical shifts in arbovirus populations. Transport of *Aedes aegypti* in ships centuries ago allowed for the transmission of yellow fever from Africa to South America, which is still happening today [60], and in the case of *Aedes albopictus,* principally through the used-car tire export trade [61]. Dengue, chikungunya, Zika, and other viral diseases have all become major threats to global public health due to this characteristic of global spread through human-facilitated means [62], which has elevated *Aedes aegypti* and *Aedes albopictus* to their current status as arguably the two most significant disease carriers on the planet. West Nile virus, a *Culex*-associated arbovirus that uses birds as an amplifying host, has also made the journey to North America [63], possibly from Africa or the Middle East, as genetic evidence suggests, and is thought to have arrived by mosquitoes on board airplanes [63]. Such disease movement is not limited to trade and tourism, as there is a growing amount of people visiting friends and relatives, pilgrimages, humanitarian and other volunteer work, and large numbers of politically and economically affected refugees, all of which contribute to the potential for infected people to carry infectious agents to other locations and infect native vectors [64].

### 4.3. Land Use Change

Several land use activities, such as deforestation or urbanization, alter arthropod habitats and create new avenues and opportunities for increased arthropod–human contact, which ultimately facilitate the spread of diseases and increase their risk. The effect of man on the planet, such as modern infrastructure, irrigation, and vast solid waste generation, favors vector growth. Finally, urbanization in impoverished areas with an insufficient water supply and rubbish disposal might encourage mosquito breeding [43,65]. For example, previous reports have indicated that there has been substantial changes in land use in several southwestern states in Nigeria and Cameroon from 2000 to 2015 for the built-up land, forestland, farmland, and mixed land in those areas [66,67]. Conversely, water body and rock outcrop land have shown less important land use changes. Other forms of land significantly reduce as the built-up land increases. These findings suggest that animals, like rats, monkeys, and rabbits, that have their natural homes in the forest and farmland could be displaced, making them dwell among humans, which implies that humans in those areas in Cameroon and Nigeria might become susceptible to vector-borne diseases, like yellow fever, Ebola, and others. In the case of JEV, although not endemic in West Africa, the processes of propagation in Asian countries are linked to changes in land use and agricultural activities [68]. Increased chances for mosquito breeding arise when the economy develops and the rice sector thrives at the price of deforestation, as rice paddy areas are regarded as an excellent condition for mosquito reproduction and growth. Furthermore, rice fields entice migrating birds, further complicating the intricate interplay of elements that characterize JEV propagation and dissemination [68].

### 4.4. Animal Reservoirs

Most arthropod-borne encephalitis viruses have animal reservoirs and perhaps amplifying hosts, which can serve as a source of infection for vectors and, in turn, humans. Rift Valley fever virus has been demonstrated in experiments to multiply in a wide range of species of mammals; however, their response to environmental infections varies greatly. Sheep, cattle, goats, and camels are the most commonly connected with severe epizootics, partly because they outnumber other possible hosts in disease-affected areas. There is now strong convincing proof that WNV spreads widely and is largely unharmful among humans, birds, horses, and a variety of other animals in Africa, Europe, many parts of Asia, and Australasia. This is based on serological investigations, virus isolation, and polymerase chain reaction sequencing using samples taken from healthy birds, horses, mosquitoes, and ticks [69]. Pigs serve as the amplifying host for JEV [68]. In addition to pigs and birds, several domesticated animals that may be sub-clinically ill, but are not likely to aid in the spread of JEV, include horses and other equids (donkeys), cattle, sheep, goats, dogs, cats, chickens, ducks, wild mammals, reptiles, and amphibians [70]. However, further studies are required to rule out alternative host animal roles in the JEV transmission cycle [71].

### 4.5. Vertical Viral Transmission and Desiccation-Resistant Eggs

Horizontal transmission of the virus occurs between mosquitos, birds, humans, and other vertebrates, but there is also a trans-generational, vertical transfer of viruses within some vector species, allowing the infectious virus to travel from adult mosquitos to their offspring [21]. Vertical viral transmission from one generation of mosquitos to the next appears to be unusual in mosquito-borne enteroviruses, but widespread in flaviviruses, and prevalent in bunyaviruses [72]. Having the capacity to pass the virus on from one mosquito generation to the next via infected eggs (vertical transmission) is a significant adaptive benefit that helps enhance the probability of virus survival in the environment, reducing the chances of the local extinction of the virus at a specific geographical focus. However, a mix of this ability with eggs that are also desiccation tolerant or resistant adds a truly strong dual framework not only for virus survival, but also for spatiotemporal spread. The ability of some mosquitoes to lay eggs that can withstand varying amounts of complete drying out and hatch when the next surges of rain, flood, or human irrigation of a garden occur, or even have staggered hatching events spread over multiple rainfall surges, is likely the most significant biological trait of all that contributed to the efficient transcontinental dissemination of *Ae. aegypti* and *Ae. albopictus*, as well as other viruses, such as DENV, CHIKV, YFV, and others that have been linked therewith [43]. How RVF virus continues to exist between epidemics that may have more than a decade between such occurrences has long been a mystery. It has been hypothesized that transovarial-infected *Aedes* eggs, that are drought-resistant and thrive for several decades, may be to blame or assist such inter-epidemic existence [73,74].

## 5. Integrated Health System: Plausible Preventive and Control Measures

The integrated health system approach, combining surveillance, vaccination, vector control, and community engagement, has been proposed to be effective in preventing most diseases through several strategies. A concerted effort combining these factors could prove to be a solution going forward.

### 5.1. Robust Surveillance

**Figure 3:**
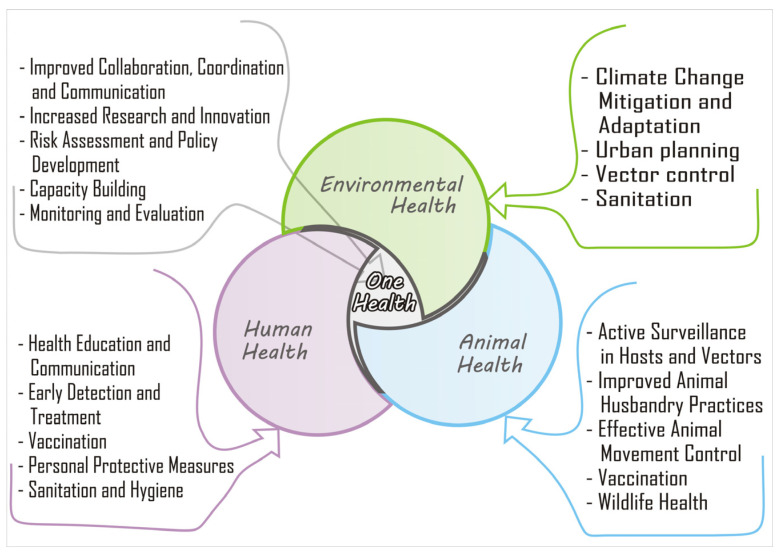
**A schematic description of the One Health triad, including human, animal, and environmental health.** The integrated “One” health system approach, combining surveillance, vaccination, vector control, and community engagement, could be effective in preventing viral encephalitis in West Africa.

As shown in Figure 3, surveillance is a cornerstone of integrated health systems in preventing VE in West Africa. Timely detection, reporting of suspected cases, and outbreaks are crucial for prompt response and control measures. Surveillance systems, such as the Integrated Disease Surveillance and Response (IDSR) strategy implemented by the World Health Organization (WHO), facilitate the collection, analysis, and dissemination of data on disease patterns, which inform public health actions. Surveillance activities for different diseases involve similar functions (detection, reporting, analysis, feedback, and action) and often use the same structures, processes, and personnel [49]. Through robust surveillance, health authorities can identify potential hotspots, monitor disease transmission dynamics, and implement interventions to prevent the spread of VE. Disease surveillance, either through passive case detection or syndromic surveillance with laboratory confirmation, is a pillar for arboviral monitoring [5]. However, arboviral infections result in a spectrum of clinical outcomes, which can be mild with only non-specific flu-like symptoms, and often managed in outpatient clinics without testing for arboviral infections. Consequently, increased virus transmission activity would be missed, since not all symptomatic infections are tested for arboviral etiology [52].

### 5.2. Vaccination

Vaccines have been proven to be effective in preventing VE diseases, which are prevalent in West Africa [73]. At the moment, there is paucity of information about vaccines against most viruses causing encephalitis in Africa. Vaccination campaigns, including routine immunization, are essential components of integrated health systems to ensure high vaccine coverage in vulnerable populations [46]. Vaccination campaigns for vaccine-preventable diseases, such as polio, have also been integrated into routine immunization programs in West Africa [47]. Vaccination, when combined with other preventive measures, can contribute to the reduction in VE cases [48]. To achieve this in West Africa, a concerted effort should be made to overcome some of the generally long-standing issues with vaccine production and development in Africa [74]. Some of these include a lack of a clear central plan or coordination, even though there are many seeming efforts in many West African countries. One other major issue is the restriction in access to adequate finance required to implement these plans. This may be resolved through collaborations with industrial giants involved in global vaccine manufacturing. More importantly, there is a need for building an array of local talents, furnished with robust capacities in genomics and functional immunology, including competencies in identifying the immune correlates of vaccine protection, vaccine design, and mass production [75]. Weak national and regional regulatory environments, and highly challenging demand dynamics are also lingering issues mitigating the development of vaccines across West Africa [76]. The lack of political will to invest in infectious disease control or otherwise is reflected in the national and regional policies per time [76]. Policy makers should actively contribute to global health by prioritizing preventive measures against several vaccine-preventable diseases.

### 5.3. Vector Control

Vector control is critical to prevent viral encephalitis in West Africa, as many of these diseases are transmitted by vectors, the climate favors the vector population and makes arthropod-borne VE prevalent. Sub-Saharan Africa hosts multiple vector-borne diseases due to the environmental conditions and high diversity of vector species [77]. For many vector-borne diseases, vector control (targeting the arthropods transmitting the disease) is highly effective in reducing transmission. For some diseases, such as dengue, vector control is the only approach currently available [49]. Previous successes indicate that thorough vector control can reduce disease incidence [78]. Unsuccessful interventions are typically attributed to several factors, including inadequate responses to the virus’ strength of transmission, insecticide resistance, expanding vector populations, expanding urban centers with poor sanitation, inadequate vector control infrastructure, and so on. [79]. To strengthen vector control, the WHO endorsed the integrated vector management strategy as a platform for combating vector-borne diseases. Integrated vector management (IVM) is a comprehensive approach that aims to optimally use resources for sustained evidence-based vector control, with the collaboration of the health sector, various agencies, and communities [50,51]. In 2004, the WHO adopted IVM globally for the control of all vector-borne diseases and has been implemented in various countries to reduce the burden of diseases, including VE [52]. Community engagement is also a critical component of vector control, where communities identify and eliminate vector breeding sites, use insecticide-treated bed nets, and adopt other preventive measures. The simultaneous use of multiple methods is now the preferred vector control strategy and forms a cornerstone of integrated vector management, a best practice framework for sustainable and cost-effective vector control. 

### 5.4. Health System Strengthening

Strengthening health systems is essential in the prevention of VE in West Africa. Weak health systems, with inadequate infrastructure, limited resources, and workforce shortages, can hinder effective disease prevention and control efforts [53]. Integrated health systems require providing diagnostic testing, treatment, and supportive care. Building laboratory diagnostics capacity, training healthcare workers, and improving healthcare infrastructure are critical components of the strengthening. In addition, strengthening supply chains, improving data management, and ensuring availability of medical supplies and drugs are crucial in West Africa.

### 5.5. Collaboration and Coordination

Collaboration and coordination among different stakeholders are essential in integrated health systems for the prevention of VE in West Africa. Viral encephalitis is a complex public health issue that requires a multisectoral approach, involving health authorities, government agencies, non-governmental organizations, community-based organizations, and other relevant partners [54]. Collaborative efforts can lead to coordinated actions, resource sharing, and efficient implementation of preventive measures. Adapting the One Health approach among these different sectors can help to address the underlying risk factors for VE. Collaboration is essential in ensuring a unified approach to preventing VE in West Africa.

## 6. Conclusions and Recommendations

Despite the potential benefits of integrated health systems in preventing VE in West Africa, there are several challenges that need to be addressed. Limited resources, including financial, human, and infrastructure, can hinder the effective implementation of integrated health systems. Inadequate funding for surveillance, vaccination campaigns, vector control, and health system strengthening can limit the capacity to prevent VE outbreaks. Additionally, workforce shortages, particularly in remote and underserved areas, can limit the delivery of essential health services. Weak health information systems and data management can also impede the timely and accurate collection, analysis, and dissemination of disease data, hampering effective response measures.

To overcome these challenges, several solutions can be implemented. Strengthening health systems through increased investments in infrastructure, workforce training, and supply chain management can improve the capacity to prevent viral encephalitis outbreaks. More important is the need to bolster efforts towards the development of vaccines against vector-borne pathogens across Africa. To achieve this, useful collaborations with scientists and industry experts from other parts of the world could be germane. Enhancing surveillance systems by investing in surveillance infrastructure, training healthcare workers, and improving data management can enhance the early detection and response to outbreaks. Increasing funding for vaccination campaigns, vector control, and community engagement programs can ensure high vaccine coverage and effective vector control measures. Promoting collaboration among different stakeholders through multi-sectoral partnerships and resource sharing can ameliorate the implementation as well. Additionally, advocacy and policy engagement can be employed to highlight the importance of integrated health systems in preventing VE and advocate for increased funding and support from government, donors, and other stakeholders.

## Data Availability

Not applicable.

## References

[1] Klein R.S. (2020). Encephalitic Arboviruses of Africa: Emergence, Clinical Presentation and Neuropathogenesis. Front. Immunol..

[2] Rezaei S.J., Mateen F.J. (2021). Encephalitis and meningitis in Western Africa: A scoping review of pathogens. Trop. Med. Int. Health.

[3] Mencattelli G., Ndione M.H.D., Rosà R., Marini G., Diagne C.T., Diagne M.M., Fall G., Faye O., Diallo M., Faye O. (2022). Epidemiology of West Nile virus in Africa: An underestimated threat. PLoS Neglected Trop. Dis..

[4] Liang G., Gao X., Gould E.A. (2015). Factors responsible for the emergence of arboviruses; strategies, challenges and limitations for their control. Emerg. Microbes Infect..

[5] Watanabe M.E. (2008). Animal Reservoirs: Harboring the Next Pandemic. BioScience.

[6] Tomori O., Oluwayelu D.O. (2023). Domestic Animals as Potential Reservoirs of Zoonotic Viral Diseases. Annu. Rev. Anim. Biosci..

[7] Weaver S.C., Reisen W.K. (2010). Present and future arboviral threats. Antivir. Res..

[8] Agboli E., Zahouli J.B.Z., Badolo A., Jöst H. (2021). Mosquito-Associated Viruses and Their Related Mosquitoes in West Africa. Viruses.

[9] Sattler R.A., Paessler S., Ly H., Huang C., Sattler R.A., Paessler S., Ly H., Huang C. (2020). Animal Models of Lassa Fever. Pathogens.

[10] Weaver S.C., Barrett A.D.T. (2004). Transmission cycles, host range, evolution and emergence of arboviral disease. Nat. Rev. Microbiol..

[11] Ogunlade S.T., Meehan M.T., Adekunle A.I., Rojas D.P., Adegboye O.A., McBryde E.S. (2021). A Review: *Aedes*-Borne Arboviral Infections, Controls and *Wolbachia*-Based Strategies. Vaccines.

[12] De Silva P.M., Marshall J.M. (2012). Factors Contributing to Urban Malaria Transmission in Sub-Saharan Africa: A Systematic Review. J. Trop. Med..

[13] Portillo A., Palomar A.M., Santibáñez P., Oteo J.A. (2021). Epidemiological Aspects of Crimean-Congo Hemorrhagic Fever in Western Europe: What about the Future?. Microorganisms.

[14] Colpitts T.M., Conway M.J., Montgomery R.R., Fikrig E. (2012). West Nile Virus: Biology, Transmission, and Human Infection. Clin. Microbiol. Rev..

[15] Akhmetzhanov A.R., Asai Y., Nishiura H. (2019). Quantifying the seasonal drivers of transmission for Lassa fever in Nigeria. Philos. Trans. R. Soc. B Biol. Sci..

[16] Caminade C., McIntyre K.M., Jones A.E. (2019). Impact of recent and future climate change on vector-borne diseases. Ann. N. Y. Acad. Sci..

[17] Ryan S.J., Carlson C.J., Mordecai E.A., Johnson L.R. (2019). Global expansion and redistribution of Aedes-borne virus transmission risk with climate change. PLoS Negl. Trop. Dis..

[18] Pandey A., Atkins K.E., Medlock J., Wenzel N., Townsend J.P., Childs J.E., Nyenswah T.G., Ndeffo-Mbah M.L., Galvani A.P. (2014). Strategies for containing Ebola in West Africa. Science.

[19] Greening G.E., Cannon J.L. (2016). Human and Animal Viruses in Food (Including Taxonomy of Enteric Viruses). Viruses Foods.

[20] Kumari R., Sharma R.S., Raina V.K., Chauhan L. (2014). Role of integrated vector management for prevention and control of Japanese encephalitis/acute encephalitis syndrome (JE/AES)—A review. J. Commun. Dis..

[21] Atkinson B., Thorburn F., Petridou C., Bailey D., Hewson R., Simpson A.J., Brooks T.J., Aarons E.J. (2017). Presence and Persistence of Zika Virus RNA in Semen, United Kingdom, 2016. Emerg. Infect. Dis..

[22] Habarugira G., Suen W.W., Hobson-Peters J., Hall R.A., Bielefeldt-Ohmann H. (2020). West Nile Virus: An Update on Pathobiology, Epidemiology, Diagnostics, Control and “One Health” Implications. Pathogens.

[23] Monath T.P., Vasconcelos P.F. (2015). Yellow fever. J. Clin. Virol..

[24] Braack L., De Almeida A.P.G., Cornel A.J., Swanepoel R., De Jager C. (2018). Mosquito-borne arboviruses of African origin: Review of key viruses and vectors. Parasites Vectors.

[25] Counotte M.J., Kim C.R., Wang J., Bernstein K., Deal C.D., Broutet N.J.N., Low N. (2018). Sexual transmission of Zika virus and other flaviviruses: A living systematic review. PLoS Med..

[26] Trivedi S., Chakravarty A. (2022). Neurological Complications of Dengue Fever. Curr. Neurol. Neurosci. Rep..

[27] Thiberville S.-D., Moyen N., Dupuis-Maguiraga L., Nougairede A., Gould E.A., Roques P., de Lamballerie X. (2013). Chikungunya fever: Epidemiology, clinical syndrome, pathogenesis and therapy. Antivir. Res..

[28] Akinsulie O.C., Adesola R.O., Bakre A., Adebowale O.O., Adeleke R., Ogunleye S.C., Oladapo I.P. (2022). Usutu virus: An emerging flavivirus with potential threat to public health in Africa: Nigeria as a case study. Front. Vet. Sci..

[29] Hayman D.T.S., Suu-Ire R., Breed A.C., McEachern J.A., Wang L., Wood J.L.N., Cunningham A.A. (2007). Evidence of Henipavirus Infection in West African Fruit Bats. PLoS ONE.

[30] Gilbert N. (2011). West Africans at risk from bat epidemics. Nature.

[31] Adamu A.M., McNabb L., Adikwu A.A., Jibril Y.J., Idoko S.I., Turaki A.U., Abalaka S.E., Edeh R.E., Egwu G.O., Adah M.I. (2022). Case report: Henipavirus sero-surveillance in horses and pigs from Northern Nigeria. Front. A J. Women Stud..

[32] Chen B.-S., Lee H.-C., Lee K.-M., Gong Y.-N., Shih S.-R. (2020). Enterovirus and Encephalitis. Front. Microbiol..

[33] Mbonye A., Wamala J., Nanyunja M., Opio A., Makumbi I., Aceng J. (2014). Ebola Viral Hemorrhagic Disease Outbreak in West Africa- Lessons from Uganda. Afr. Health Sci..

[34] Buseh A.G., Stevens P.E., Bromberg M., Kelber S.T. (2015). The Ebola epidemic in West Africa: Challenges, opportunities, and policy priority areas. Nurs. Outlook.

[35] Rohde R.E., Rupprecht C.E. (2019). Update on lyssaviruses and rabies: Will past progress play as prologue in the near term towards future elimination?. Fac. Rev..

[36] Fooks A.R., Cliquet F., Finke S., Freuling C., Hemachudha T., Mani R.S., Müller T., Nadin-Davis S., Picard-Meyer E., Wilde H. (2017). Rabies. Nat. Rev. Dis. Prim..

[37] Rupprecht C.E., Mani R.S., Mshelbwala P.P., Recuenco S.E., Ward M.P. (2021). Rabies in the Tropics. Curr. Trop. Med. Rep..

[38] Johns Hopkins Medicine Encephalitis. https://www.hopkinsmedicine.org/health/conditions-and-diseases/encephalitis#:~:text=The%20most%20common%20causes%20of,enteroviruses%2C%20which%20cause%20gastrointestinal%20illness.

[39] Perlejewski K., Rydzanicz M., Pawełczyk A., Cortѐs K.C., Osuch S., Paciorek M., Dzieciątkowski T., Radkowski M., Laskus T. (2020). Next-generation sequencing in the diagnosis of viral encephalitis: Sensitivity and clinical limitations. Sci. Rep..

[40] Tamura K. (1992). Estimation of the number of nucleotide substitutions when there are strong transition-transversion and G+C-content biases. Mol. Biol. Evol..

[41] Saitou N., Nei M. (1987). The neighbor-joining method: A new method for reconstructing evolutionary trees. Mol. Biol. Evol..

[42] Felsenstein J. (1985). Confidence limits on phylogenies: An approach using the bootstrap. Evolution.

[43] Kilpatrick A.M., Randolph S.E. (2012). Drivers, dynamics, and control of emerging vector-borne zoonotic diseases. Lancet.

[44] Mordecai E.A., Ryan S.J., Caldwell J.M., Shah M.M., LaBeaud A.D. (2020). Climate change could shift disease burden from malaria to arboviruses in Africa. Lancet Planet. Health.

[45] Medlock J.M., Leach S.A. (2015). Effect of climate change on vector-borne disease risk in the UK. Lancet Infect. Dis..

[46] Paz S., Malkinson D., Green M.S., Tsioni G., Papa A., Danis K., Sirbu A., Ceianu C., Katalin K., Ferenczi E. (2013). Permissive Summer Temperatures of the 2010 European West Nile Fever Upsurge. PLoS ONE.

[47] Kinney R.M., Huang C.Y.-H., Whiteman M.C., Bowen R.A., Langevin S.A., Miller B.R., Brault A.C. (2006). Avian virulence and thermostable replication of the North American strain of West Nile virus. J. Gen. Virol..

[48] Kilpatrick A.M., Meola M.A., Moudy R.M., Kramer L.D. (2008). Temperature, Viral Genetics, and the Transmission of West Nile Virus by Culex pipiens Mosquitoes. PLoS Pathog..

[49] Paz S., Albersheim I. (2008). Influence of Warming Tendency on Culex pipiens Population Abundance and on the Probability of West Nile Fever Outbreaks (Israeli Case Study: 2001–2005). EcoHealth.

[50] Landesman W.J., Allan B.F., Langerhans R.B., Knight T.M., Chase J.M., Ferenczi M., Beckmann C., Warner S., Loyn R., O’riley K. (2007). Inter-Annual Associations Between Precipitation and Human Incidence of West Nile Virus in the United States. Vector-Borne Zoonotic Dis..

[51] Takeda T., Whitehouse C.A., Brewer M., Gettman A.D., Mather T.N. (2003). Arbovirus surveillance in Rhode Island: Assessing potential ecologic and climatic correlates. J. Am. Mosq. Control Assoc..

[52] Campbell G.L., Marfin A.A., Lanciotti R.S., Gubler D.J. (2002). West Nile virus. Lancet Infect. Dis..

[53] Trawinski P., MacKay D. (2008). Meteorologically Conditioned Time-Series Predictions of West Nile Virus Vector Mosquitoes. Vector-Borne Zoonotic Dis..

[54] Meegan J.M., Bailey C.L., Monath T.P. (1988). Rift Valley fever. The Arboviruses: Epidemiology and Ecology.

[55] Glancey M.M., Anyamba A., Linthicum K.J., Clements A.C., Pfeiffer D.U., Martin V., Pittiglio C., Best N., Thiongane Y., Jeanmaire E.M. (2015). Epidemiologic and Environmental Risk Factors of Rift Valley Fever in Southern Africa from 2008 to 2011. Vector-Borne Zoonotic Dis..

[56] Chretien J.P., Anyamba A., Bedno S.A., Breiman R.F., Sang R., Sergon K., Powers A.M., Onyango C.O., Small J., Tucker C.J. (2007). Drought-associated chikungunya emergence along coastal East Africa. Am. J. Trop. Med. Hyg..

[57] Aliyu A.A., Amadu L. (2017). Urbanization, cities, and health: The challenges to Nigeria—A review. Ann. Afr. Med..

[58] Bongaarts J. (2009). Human population growth and the demographic transition. Philos. Trans. R. Soc. B Biol. Sci..

[59] United Nations (2013). World Population Prospects, the 2012 Revision.

[60] Bryant J.E., Holmes E.C., Barrett A.D.T. (2007). Out of Africa: A Molecular Perspective on the Introduction of Yellow Fever Virus into the Americas. PLoS Pathog..

[61] Gratz N.G. (2004). Critical review of the vector status of Aedes albopictus. Med. Vet. Éntomol..

[62] Benedict M.Q., Levine R.S., Hawley W.A., Lounibos L.P., Lwande O.W., Obanda V., Lindström A., Ahlm C., Evander M., Näslund J. (2007). Spread of The Tiger: Global Risk of Invasion by The Mosquito *Aedes albopictus*. Vector-Borne Zoonotic Dis..

[63] Brown C.R., O’Brien V.A. (2011). Are Wild Birds Important in the Transport of Arthropod-Borne Viruses?—¿Son Las Aves Silvestres Importantes Para El Transporte de Virus Transmitidos Por Artrópodos?. Ornithol. Monogr..

[64] Charrel R.N., de Lamballerie X., Raoult D. (2007). Chikungunya Outbreaks—The Globalization of Vectorborne Diseases. N. Engl. J. Med..

[65] Poulakou G., Plachouras D. (2016). Planet’s population on the move, infections on the rise. Intensiv. Care Med..

[66] Ewane E.B. (2021). Land use land cover change and the resilience of social-ecological systems in a sub-region in South west Cameroon. Environ. Monit. Assess..

[67] Izah L.N., Majid Z., Ariff M.F.M., Mohammed H.I. (2018). Determining land use change pattern in southern Nigeria: A comparative study. IOP Conf. Ser. Earth Environ. Sci..

[68] Solomon T. (2006). Control of Japanese Encephalitis—Within Our Grasp?. N. Engl. J. Med..

[69] Buckley A., Dawson A., Moss S.R., Hinsley S.A., Bellamy P.E., Gould E.A. (2003). Serological evidence of West Nile virus, Usutu virus and Sindbis virus infection of birds in the UK. J. Gen. Virol..

[70] OIE Technical Disease Cards (2013). Japanese Encephalitis. http://www.oie.int/fileadmin/Home/eng/Animal_Health_in_the_World/docs/pdf/Disease_cards/JAPANESE_ENCEPHALITIS.pdf.

[71] Oliveira A.R.S., Cohnstaedt L.W., Cernicchiaro N. (2018). Japanese Encephalitis Virus: Placing Disease Vectors in the Epidemiologic Triad. Ann. Éntomol. Soc. Am..

[72] García-Carrasco J.-M., Muñoz A.-R., Olivero J., Segura M., Real R. (2022). Mapping the Risk for West Nile Virus Transmission, Africa. Emerg. Infect. Dis..

[73] Reisen W.K., Fang Y., Martinez V.M. (2006). Effects of temperature on the transmission of west nile virus by Culex tarsalis (Diptera: Culicidae). J. Med. Entomol..

[74] Makenga G., Bonoli S., Montomoli E., Carrier T., Auerbach J. (2019). Vaccine Production in Africa: A Feasible Business Model for Capacity Building and Sustainable New Vaccine Introduction. Front. Public Health.

[75] Sullivan N.J., Martin J.E., Graham B.S., Nabel G.J. (2008). Correlates of protective immunity for Ebola vaccines: Implications for regulatory approval by the animal rule. Nat. Rev. Genet..

[76] Oladipo H.J., Tajudeen Y.A., Oladunjoye I.O., Yusuff S.I., Yusuf R.O., Oluwaseyi E.M., AbdulBasit M.O., Adebisi Y.A., El-Sherbini M.S. (2022). Increasing challenges of malaria control in sub-Saharan Africa: Priorities for public health research and policymakers. Ann. Med. Surg..

[77] Andrade C.C., Maharaj P.D., Reisen W.K., Brault A.C. (2011). North American West Nile virus genotype isolates demonstrate differential replicative capacities in response to temperature. J. Gen. Virol..

[78] Meyer R.P., Hardy J.L., Reisen W.K. (1990). Diel Changes in Adult Mosquito Microhabitat Temperatures and Their Relationship to the Extrinsic Incubation of Arboviruses in Mosquitoes in Kern County, California. J. Med. Éntomol..

[79] Ruiz M.O., Chaves L.F., Hamer G.L., Sun T., Brown W.M., Walker E.D., Haramis L., Goldberg T.L., Kitron U.D. (2010). Local impact of temperature and precipitation on West Nile virus infection in Culex species mosquitoes in northeast Illinois, USA. Parasites Vectors.

